# Communicating Community-Based Public Health Surveillance: Lessons from Profiling Public Risk Perceptions of COVID-19 Wastewater Monitoring

**DOI:** 10.3390/ijerph22121782

**Published:** 2025-11-25

**Authors:** Youllee Kim

**Affiliations:** Department of Communication Studies, University of Denver, 298 Sturm Hall, 2000 E. Asbury Ave., Denver, CO 80208, USA; youllee02@gmail.com

**Keywords:** health messages, audience profiles, COVID-19, wastewater monitoring, communal coping orientation, COVID-19 misinformation

## Abstract

Wastewater monitoring is a well-established form of community-based public health surveillance technology that gained renewed attention during the COVID-19 pandemic as an early warning system for SARS-CoV-2 infection trends. For monitoring data to be effectively translated into public health action, however, communication strategies that address public risk perceptions and foster cooperation are essential. This study focuses on wastewater monitoring in the context of COVID-19 and provides an evidential basis for developing targeted public health messages by segmenting the population into risk perception profiles. A survey of 332 Colorado residents was analyzed using latent class analysis (LCA), revealing four profiles: the worrisome (48%), the practical (19%), the community-oriented (11%), and the minimally concerned (22%). LCA with covariate analysis showed that communal coping orientation, belief in misinformation, and attitudes and knowledge of wastewater monitoring, along with age, education, and political ideology, were associated with these profiles. Findings highlight how communication strategies for community-based public health surveillance can be tailored to different population subgroups.

## 1. Introduction

### 1.1. Communicating Community-Based Public Health Surveillance: Lessons from Wastewater Monitoring Risk Profiling

Wastewater monitoring, also referred to as wastewater-based epidemiology or wastewater surveillance, is a form of community-based public health surveillance that provides early warnings for health threats through non-invasive, anonymous community-level sampling from wastewater [1]. During the COVID-19 pandemic, wastewater surveillance offered critical insights when testing resources were scarce, enabling more proactive interventions [2,3]. Despite the promise of wastewater monitoring, the public’s understanding of this public health tool remains limited [4]. Moreover, health privacy violations and community stigmatization have been identified as potential risks shaping public acceptance of this surveillance tool [5,6]. To ensure wastewater monitoring and other community-based surveillance approaches are both effective and trusted, communication strategies must address public concerns about potential risks, such as privacy and data misuse, while emphasizing their public health benefits. This study adopts an exploratory, person-centered approach to identify distinct risk perception profiles related to wastewater monitoring for COVID-19—groups of individuals characterized by differing perceptions of the associated risks. By uncovering meaningful audience typologies, the study aims to inform the design of tailored communication strategies that bridge the gap between public health practice and public understanding, thereby supporting the broader integration of community-based health surveillance into public health communication.

### 1.2. Science and Utility of Wastewater Monitoring of Infectious Diseases

Wastewater monitoring has a long history in public health, dating back to the 1800s when it helped trace cholera outbreaks in London [7]. Over time, it expanded to detect pathogens like *Salmonella typhi* (which causes typhoid) and poliovirus [8,9], playing a key role in global polio eradication efforts since the 1970s [10]. More recently, it has been used to monitor population-level health trends and behaviors, such as disease transmission and drug use [2,11]. During the COVID-19 pandemic, wastewater surveillance emerged as a critical tool to track SARS-CoV-2, especially when clinical testing was limited by supply shortages and low public participation [12,13].

Detecting SARS-CoV-2 RNA in wastewater offers a time-sensitive, non-invasive, and less biased approach that supplements clinical diagnosis [14]. When infected, most people shed SARS-CoV-2 in their feces regardless of whether they are symptomatic or asymptomatic [15] and may begin shedding before any symptoms appear [16]. Since the samples are collected from wastewater treatment facilities and sewers, wastewater monitoring is a non-invasive method for tracking the occurrence and prevalence of SARS-CoV-2 infection at the population level [17]. As wastewater samples are taken from communal waste streams that draw from all contributing individuals with equal probability, wastewater monitoring of COVID-19 is also considered a less biased method that is independent of the population’s healthcare access or testing availability [18]. Wastewater monitoring is also well-suited for long-term surveillance, as clinical testing is resource-intensive, requiring significant investment in reagents, equipment, and personnel [19]. By contrast, wastewater monitoring can screen large populations with a single sample, making it a more cost-effective solution over time despite initial infrastructure costs [19].

Increasing episodes of epidemics and pandemics, including SARS-CoV-1, H1N1, MERS, Ebola, and SARS-CoV-2, have heightened the urgency for global public health agencies to enhance their disease surveillance capabilities [20]. In the U.S., the Centers for Disease Control and Prevention (CDC) and the U.S. Department of Health and Human Services have also developed a National Wastewater Surveillance System (NWSS), collaborating with the U.S. Environmental Protection Agency, the U.S. Department of Homeland Security, and other partners [21,22].

At the time of data collection (June–July 2023) for the current study, the United States had transitioned from emergency COVID-19 response to a long-term surveillance stage [21]. Wastewater monitoring for SARS-CoV-2 had been introduced nationwide through the NWSS but was still in various stages of implementation across states [23,24]. As wastewater monitoring programs continue, it is critical to ensure that the system remains adaptable, addressing not only ongoing challenges from infectious diseases such as COVID-19, influenza, and respiratory syncytial virus (RSV), but also expanding to broader applications, including chronic conditions and drug use [25]. Importantly, long-term wastewater monitoring should be widely acceptable to the public, as public support facilitates the integration of this complementary surveillance system into long-term health infrastructure rather than limiting it to short-term crisis response. Public understanding of wastewater monitoring can also shape how individuals respond to public health policies derived from wastewater monitoring data. As uncertainties and associated risk perceptions play significant roles in determining public acceptance of technologies [26,27], the following section explores the public’s risk perceptions of wastewater monitoring.

### 1.3. Public’s Risk Perceptions of Wastewater Monitoring

Effective public health communication likely depends on its capacity to identify and predict how segments of the population differ in their risk beliefs about wastewater monitoring [28,29]. One possible risk perception involves uncertainty about the reliability of the wastewater monitoring technology. Although various scientific studies and guidelines have worked to establish methods for enhancing the accuracy and reliability of wastewater monitoring [2], many members of the public remain uncertain about the dependability of these new technologies. This uncertainty may also raise questions about the cost-effectiveness of wastewater monitoring. Given that the public is not generally exposed to wastewater monitoring in daily life [30], it is understandable that there may be a lack of trust or understanding. This unfamiliarity breeds uncertainty [31], which can amplify perceived risks. If people do not fully grasp how technology works or how data is collected and analyzed, they may worry about its potential inaccuracies or limitations.

Beyond concerns about technological reliability and cost-effectiveness, privacy is a significant issue for many individuals. Wastewater surveillance typically does not require informed consent when samples are collected from public infrastructure, as individuals are not identifiable and thus not directly harmed [6,32,33]. However, concerns may still arise over data ownership and access. When the purpose and scope of monitoring are not clearly communicated, fears about potential misuse or unauthorized access to health information may reduce public support. Worries also extend to the potential use of wastewater data for monitoring illegal activities, such as drug use [34,35]. Although ethical guidelines recommend using such data only for identifying population-level trends to inform public health policies [36], fears of individual surveillance—particularly by law enforcement—could undermine public trust and support [37].

Another concern is that wastewater monitoring data could stigmatize individuals or communities. Although individuals are not identifiable, such data may still lead to communities being unfairly labeled as sources of infection, fostering discrimination and social stigma [38]. This was evident during the COVID-19 pandemic, when higher infection rates in certain areas fueled racism, scapegoating, and unequal treatment [39,40]. Additionally, there might be concerns about how wastewater monitoring data could influence policy decisions at the community level [41]. For instance, drastic actions like business and school closures or the unequal distribution of resources were implemented during the pandemic [42], and similar measures could potentially be taken based on wastewater data. Addressing these risks through clear policy guidelines and safeguards is essential for sustaining wastewater monitoring as a trusted public health tool [6].

### 1.4. Profiles of Risk Perceptions and Their Covariates

This study employs Latent Class Analysis (LCA; [43]) to segment the public based on their risk perceptions of wastewater monitoring for COVID-19. As the first study to apply audience segmentation in this context, it uses LCA to identify homogeneous subgroups within a diverse population [44]. LCA is well-suited for this purpose for two key reasons. First, risk perceptions are multidimensional and interrelated [45,46]; segmenting audiences based on a single risk belief oversimplifies this complexity. LCA allows for classification based on multiple risk perceptions, capturing the complexity of public perceptions on wastewater monitoring. Second, LCA provides a means to examine population heterogeneity when existing theories do not clearly outline how to classify the audience based on relevant variables. Given this study’s exploratory nature in understanding public risk perceptions about wastewater monitoring for COVID-19, LCA can help identify the optimal number of subgroups that represent the population [43].

Research Question 1 (RQ 1): Is there a latent class structure that adequately represents the heterogeneity in the general public’s risk perceptions about wastewater monitoring of COVID-19?

Furthermore, LCA with covariates allows researchers to assess whether certain variables influence the likelihood of subgroup membership [29]. While prior studies have examined factors shaping concerns about wastewater monitoring, it remains unclear how these variables relate to the audience profiles identified in RQ1. Given that infectious diseases like COVID-19 pose both individual and collective health threats, people may differ in how they believe their communities should respond [47]. To explore this, the study includes communal coping orientation, defined as the extent to which individuals view the stressor as jointly owned and managed [48], as a potential covariate. Second, belief in COVID-19 misinformation is examined, as prior research suggests such beliefs can shape support for public health measures [49]. Third, knowledge about wastewater monitoring is included, given that public understanding of this method remains limited and may influence acceptance [4,5]. Finally, attitudes toward wastewater monitoring are assessed, as more favorable attitudes are associated with lower concern and greater support for its use in tracking COVID-19 [50].

Research question 2 (RQ2): Do communal coping orientation, misinformation beliefs, basic knowledge about wastewater monitoring, and attitudes toward wastewater monitoring relate to audience profiles of concern regarding wastewater monitoring of COVID-19?

Lastly, demographic factors may be critical for making decisions about which audience segment to target and through which means to target them.

Research question 3 (RQ3): Do demographic variables (i.e., age, education level, gender, race/ethnicity, and political ideology) relate to audience profiles of concerns about wastewater monitoring for COVID-19?

## 2. Methods

### 2.1. Participants and Procedures

Upon receiving approval from the university’s institutional review board, participants were recruited via Amazon’s Mechanical Turk (*n* = 174) and Prolific (*n* = 162). These are two widely used online crowdsourcing platforms that allow researchers to post studies, which participants voluntarily complete in exchange for monetary compensation [51,52]. The survey was conducted between 2 June and 1 July 2023, as part of a large project to understand the public perceptions of COVID-19 messaging and prevention measures in Colorado, United States. The state was chosen due to its strong commitment to sustainable surveillance and academic partnerships. Since August 2020, the Colorado Department of Public Health and Environment (CDPHE) has collaborated with academic and utility partners to develop a statewide wastewater surveillance system. Initially focused on COVID-19, the program has expanded to monitor other respiratory viruses, including influenza A/B, RSV, and enterovirus D68, with samples collected twice weekly and data regularly updated [53].

Participants first provided consent, confirmed their Colorado residency, and selected the wastewater utility area closest to their home. They then completed measures assessing how their community has been handling COVID-19 (i.e., communal coping orientation) and their belief in COVID-19 misinformation. Following this, they read an informational message about wastewater monitoring and responded to questions about risk perceptions, knowledge, and attitudes toward the practice. The survey concluded with sociodemographic items. The informational message provided to the participants and all survey items are included in the Supplementary Material S1. Participants received $6 for their time, with most completing the survey in under 30 min (*M* = 20.41, *SD* = 15.49). Eight individuals who failed an attention check and six who completed the survey in under five minutes were excluded, resulting in a final sample of 322 Colorado residents. Of the 67 listed utilities, participants identified 54 as serving or located near their residence. Table 1 reports detailed demographic information of the participants and compares that with 2023 U.S. Census Bureau data for Colorado [54,55,56,57].

### 2.2. Measurement

#### 2.2.1. Latent Class Indicators

Participants’ concerns with wastewater monitoring for COVID-19 were assessed on 5-point scales (1 = *strongly disagree* to 5 = *strongly agree*) and collapsed into two categories for the LCA. Responses of *strongly disagree* and *somewhat disagree* were coded as “not concerned,” whereas *neither disagree nor agree*, *somewhat agree*, and *strongly agree* were coded as “concerned or unsure.” The “unsure” category reflects participants who selected *neither disagree nor agree*. This coding approach treats those who strongly or somewhat disagreed as not very concerned about wastewater monitoring, while grouping all other responses as concerned or unsure. The decision to include “neither disagree nor agree” with the concerned category reflects the interpretation that uncertainty may indicate mixed attitudes or susceptibility to misinformation [58,59]. Table 2 presents the descriptive statistics for a total of eight latent class indicators. Detailed distributions of participants’ responses on the 5-point scales assessing concerns about wastewater monitoring for COVID-19 are provided in the Supplementary Material S2.

#### 2.2.2. Covariates

All variables were based on 5-point scales (1 = *strongly disagree* to 5 = *strongly agree*) and averaged, unless otherwise stated. Across items for covariates, there were a total of two missing observations. A missing observation for an individual on a given variable was replaced with the mean for non-missing observations for that variable [60]. A confirmatory factor analysis of multi-item scales—communal coping orientation, COVID-19 misinformation, and wastewater monitoring attitude—was estimated with maximum likelihood. All factors were allowed to covary, but errors were not. The model showed reasonable goodness of fit: *χ*^2^ (*df* = 127, *N* = 322) = 364.92, *p* < 0.001, CFI = 0.93, GFI = 0.89, SRMR = 0.08.

Communal coping was assessed by asking participants to think about how they and their community have been handling COVID-19 (Adapted from [47,61]). Three items measured the appraisal dimensions (e.g., “When I think about COVID-19, I mostly think about how it is my community’s health issue that we face together), and the other three items measured the *action* dimension (e.g., “It is my community’s responsibility to prevent the spread of COVID-19”). As per [62], we created a composite score of communal coping by combining the appraisal and action items such that higher scores indicated stronger communal coping orientations for COVID-19 (*α* = 0.84).

COVID-19 misinformation beliefs were assessed with five items (e.g., “Coronavirus is probably a hoax”) adapted from [49]. Responses were averaged into one score such that higher scores indicated stronger COVID-19 misinformation beliefs (*α* = 0.89).

Knowledge about COVID-19 wastewater monitoring was measured with three true/false questions about the information presented in the message. Participants’ scores were calculated as the total number of correct responses. Because the items for knowledge were a series of fact-based true and false questions, it is inappropriate to use alpha as a measure of internal consistency.

Attitudes toward COVID-19 wastewater monitoring were measured with four items, including “I like the idea of COVID-19 monitoring in wastewater,” and “I think COVID-19 monitoring in wastewater is desirable.” Responses were averaged into one score such that higher scores indicated stronger positive attitudes toward COVID-19 wastewater monitoring (*α* = 0.96).

At the end of the survey, participants answered demographic questions, such as age, education level, gender, race/ethnicity, as well as political ideology.

## 3. Results

### 3.1. Descriptive Statistics

As shown in Table 2, over 70% of participants were concerned or unsure that wastewater monitoring for COVID-19 may not be cost-effective (73.3%) and may be used to support drastic measures such as shutting down businesses and schools (72%). More than 60% were concerned or unsure that wastewater monitoring may not provide accurate early warnings of potential COVID-19 outbreaks (68.3%), may contribute to inequitable resource allocation in responding to COVID-19 (68%), and may be used to imply that certain individuals or communities are responsible for spreading the virus (65.8%). Participants were least concerned about privacy invasion; nevertheless, more than half were concerned or unsure that wastewater monitoring may allow personal health information to be accessible to unauthorized entities (57.1%) or may be used to trace illegal drug use (56.2%).

On average, participants slightly leaned toward a communal coping orientation (*M* = 3.71, *SD* = 0.79). The mean level of COVID-19 misinformation belief was 2.36 (*SD* = 1.15) on a 1–5 scale, suggesting that participants generally tended to disagree with misinformation statements about COVID-19, though responses showed some variability. Most participants correctly answered at least two out of three questions assessing knowledge about wastewater monitoring for COVID-19 (*M* = 2.39, *SD* = 0.71). Attitudes toward COVID-19 wastewater monitoring were generally positive (*M* = 3.76, *SD* = 1.08).

### 3.2. Latent Class Analysis

RQ1 asked whether there exists a latent class structure that adequately represents the heterogeneity in the public’s concerns about COVID-19 wastewater monitoring. To address RQ1, PROC LCA was used in SAS 9.4 to calculate the fit indices for two- to seven-class models. Each test was run using 100 random starting values. LCA follows an iterative maximum likelihood estimation process, sequentially testing models with different numbers of classes to determine the best class structure [63]. No single model outperformed the others on all the estimated parameters, which is not uncommon [43]. Along with the fit statistics, parsimony, interpretability, and percentage of participants in each class were examined to guide model selection. The two- and three-class models did not fit the criteria of having a G^2^ value less than the degrees of freedom. The AIC estimates were minimized at the seven-class model, whereas the BIC and adjusted BIC estimates were minimized at the four-class model. The four-class model was selected considering that the BIC estimating procedure emphasizes parsimony [43].

In addition to evaluating model fit, classification diagnostics need to be examined. Generally, higher entropy values are preferred, with values above 0.80 considered acceptable [64]. The average latent class posterior probability indicates how accurately the model assigns individuals to their most likely class, with values greater than 0.90 reflecting ideal classification quality [65]. Although recommendations regarding class size and proportion have become more flexible, it has been recommended that each class contains at least 50 individuals and represents no less than 5% of the total sample [65]. The four-class model also had higher entropy values and higher average latent class posterior probability than the seven-class model. The smallest class in the four-class model included 57 individuals, representing 11.37% of the total sample (See Table 3).

Table 4 shows the item-response probabilities for the four-class model and the likelihood of reporting risk perceptions about wastewater monitoring for COVID-19 within a class (i.e., the responses coded as 2 in Table 2). The four models were labeled as the *worrisome* (48%), the *minimally concerned* (22%), the *practical* (19%), and the *community-oriented* group (11%) based on the distinctive features of the concerns shared by the group. The percentages in parentheses represent the estimated proportion of participants most likely belonging to each class.

### 3.3. Covariate Analysis

RQ2 explored whether communal coping orientation for COVID-19, COVID-19 misinformation belief, and attitude toward, as well as knowledge of wastewater monitoring for COVID-19, relate to membership in the classes of risk beliefs about wastewater monitoring for COVID-19. All of the covariates were statistically significantly associated with class membership at *p* < 0.05.

RQ3 explored whether demographic variables were related to membership in the latent classes of risk beliefs about wastewater monitoring for COVID-19. Covariates included age (measured in years), education (ranging from 1 = *less than high school* to 6 = *graduate degree*), gender (1 = *female*, 0 = *non-female*), race (1 = *White*, 0 = *non-White*), and political ideology (1 = *extremely liberal* to 5 = *extremely conservative*). Of these variables, age, education, and political ideology showed statistically significant associations at *p* < 0.05.

Table 5 shows the results of the covariate analysis, with the *minimally concerned* as the referent class. Of note, although several covariates showed statistically significant effects, the confidence intervals for some estimates overlapped across groups, indicating that the differences among the practical, community-oriented, and worrisome groups were not always statistically distinct. This pattern suggests that while the covariates meaningfully distinguish the minimally concerned group from the other classes, comparisons among the remaining groups should be interpreted with caution.

## 4. Discussion

This study explored risk perception profiles related to wastewater monitoring for COVID-19 and examined covariates associated with those profiles. Through latent class analysis (LCA), we identified four distinct risk profiles, each reflecting a different pattern of concerns about this health surveillance technology. These profiles provide an evidential basis for tailoring communication strategies, ensuring that public health messages can address the specific concerns of different population subgroups. Beyond wastewater monitoring, the findings highlight how audience segmentation can inform other community-based health surveillance initiatives, where effectiveness depends not only on technical performance but also on public trust.

The *worrisome* group (48%) showed high concerns across all risks, including cost, privacy, and stigma. Communication strategies targeting this group should address these issues comprehensively rather than in isolation, emphasizing transparency, fairness, and ethical safeguards. Importantly, these concerns are not unique to wastewater monitoring, but mirror challenges faced in other community-based health surveillance initiatives, underscoring the need for trust-building communication across surveillance contexts.

The *practical* group (19%) was mainly concerned about the accuracy and cost-effectiveness of wastewater monitoring as well as the possible inequitable resource allocation based on the monitoring data. As highlighted in several studies (e.g., [32,53]), effective communication about community-based health surveillance must address both the technical foundations and the ethical use of data in public health decision-making. The present study extends this work by showing that such concerns are especially salient for the *practical* group.

The *community-oriented* group (11%) showed concerns about the potential consequences of wastewater monitoring data on the communities, such as stigmatization, discrimination, and potential misuse (e.g., tracing illicit drug use). Communication strategies tailored to this group should therefore emphasize the ethical boundaries of wastewater monitoring, community protection against misuse, and safeguards to ensure that surveillance serves public health without reinforcing stigma or inequities.

The *minimally concerned* group (22%) did not show any particular concern toward wastewater monitoring of COVID-19. A key communication challenge with this group is that individuals with low-risk beliefs are often the least motivated to seek information and acquire new knowledge [66]. When targeting the *minimally concerned* group, the interventions should make sure that a low level of concern does not translate into indifference or disengagement from public health surveillance.

All of the psychological factors (i.e., communal coping orientation for COVID-19, COVID-19 misinformation belief, and attitude toward as well as knowledge of wastewater monitoring for COVID-19) proposed in RQ2 were statistically significant covariates of class membership. Individuals with a stronger communal coping orientation were more likely to be in the *minimally concerned* group than in the *practical* group. While decisions about message framing should be made cautiously and aligned with broader research evidence, these findings suggest that public health campaigns may benefit from emphasizing communal responsibility and shared benefits to address public concerns and sustain engagement with community-based public health surveillance. Individuals with stronger COVID-19 misinformation beliefs were more likely to be in the *community-oriented* and *worrisome* groups than the *minimally concerned* group. Although correcting misinformation is inherently challenging—particularly in the context of infectious diseases, where beliefs tend to be especially resistant to change [49,67]—researchers have proposed several strategies. One such approach involves reframing public health messages to shift focus away from human causes of infection and instead emphasize pathogens as the primary threat, using linguistic agency [67]. For example, ref. [68] demonstrated that when health messages assign agency to viruses and vaccines rather than to humans, the virus is perceived as more serious and the vaccine as more effective. In light of this, public health messages could assign linguistic agency to viruses (e.g., COVID-19 affects millions of people) instead of humans (e.g., “Millions of people contract COVID-19”) to increase perceived severity of COVID-19. Additionally, public health messaging may benefit from attributing agency to the intervention itself (e.g., “COVID-19 wastewater monitoring data can guard people”) rather than to individuals (e.g., “People can guard themselves using COVID-19 wastewater monitoring data”) to enhance the perceived effectiveness of the community-based public health surveillance.

Higher knowledge was associated with lower odds of belonging to the *worrisome* or *community-oriented groups* compared to the *minimally concerned* group, suggesting the importance of incorporating educational content into public health messaging to enhance public understanding of how community-based public health surveillance works and to proactively address common misconceptions. Furthermore, positive attitudes toward wastewater monitoring were also significantly associated with class membership: in comparison to those in the *minimally concerned* group, participants who reported higher positive attitudes toward wastewater monitoring for COVID-19 had lower odds of being in the *practical*, the *community-oriented*, and the *worrisome* groups. Having minimal concern about wastewater monitoring is not problematic. However, low concern combined with low positive attitudes may reduce support. Building on the findings related to the communal coping orientation, the *minimally concerned* group could be targeted with messages that frame public health threats as shared challenges requiring collective action, while also emphasizing how community-based surveillance tools such as wastewater monitoring can help communities respond effectively.

Demographic factors also mattered. Older participants had lower odds of belonging to the *worrisome* group compared to the *minimally concerned* group, indicating that communication efforts directed toward older populations could focus on addressing any lingering uncertainties. Education lowered the odds of belonging to the *practical group* relative to the *minimally concerned* group, suggesting those with less education may question the effectiveness of surveillance tools. Communication for this population should be clear and accessible, explaining how wastewater monitoring works, why it is cost-effective, and addressing fairness concerns. A conservative political orientation increased the likelihood of membership in the *practical* and *worrisome* groups, suggesting that communication strategies should focus on enhancing trust and acceptance by emphasizing tangible benefits, transparency in government decision-making, and privacy protections.

### Limitations and Future Research Directions

Interpretation of the findings should be considered in relation to the strengths and weaknesses of the study design. First, it is important to note that this study focused on public perceptions of wastewater monitoring for COVID-19. Public perceptions of wastewater monitoring may vary depending on factors such as the perceived severity, transmissibility, and familiarity of a disease; therefore, respondents’ risk perceptions in this study may not fully generalize to the use of wastewater monitoring for other purposes and may depend on the specific health context. Despite this limitation, the methodological approach and findings provide valuable insights that can inform future research on public perceptions toward wastewater monitoring for other infectious diseases.

Second, while the eight latent class indicators capture key aspects of public concern, they are not all-encompassing. In line with efforts to expand wastewater monitoring for COVID-19 and other infectious diseases, it is essential to examine additional concerns—such as mistrust in government (e.g., [69])—and psychological barriers, particularly affective barriers (e.g., [46]), that may contribute to public reluctance to support wastewater monitoring. Relatedly, the present study dichotomized response items (1–2 not concerned vs. 3–5 concerned) to identify patterns of concern regarding COVID-19 wastewater monitoring. Although this approach enhances interpretability, we acknowledge that it may limit the granularity of attitudinal variation. We also note our decision to include a no-opinion response option (i.e., “neither disagree nor agree”) in the analysis. While such options can prevent forced responses and capture uncertainty, research suggests they may also discourage respondents from fully engaging in the cognitive effort needed to report their true opinions [58]. By grouping “neither disagree nor agree” responses with those expressing concern, this study aimed to capture the possibility that uncertainty reflects mixed attitudes or susceptibility to misinformation [59]. Nevertheless, future research should examine how neutral responses relate to underlying uncertainty and whether alternative survey designs (e.g., excluding no-opinion options) could further improve data quality and interpretability.

Third, platforms like Amazon MTurk and Prolific often underrepresent lower-income and less-educated individuals due to barriers like limited internet access and digital literacy [70]. Although the study sample included a diverse range of participants, it was not fully representative of Colorado’s demographic composition. Certain racial and ethnic groups (e.g., Hispanic/Latino and Black/African American) were underrepresented, while individuals with higher education levels were overrepresented. The study sample also included fewer high-income individuals compared to the state population. These limitations warrant caution in generalizing the findings and highlight the need for alternative or complementary recruitment strategies to enhance representativeness. The sample’s restriction to Colorado residents further limits generalizability. While the study offers valuable insights into public concerns about COVID-19 wastewater monitoring, future research using a nationally representative sample is needed to confirm broader applicability.

Lastly, due to the choice of a cross-sectional design, this study’s findings provide only a snapshot of a dynamic process. One way to address this limitation in future research is to employ a longitudinal design (e.g., latent transition analysis; [71]) that measures the indicators and predictors of audience profiles at multiple time points. Future research could also examine whether changes in covariates, such as knowledge of COVID-19 wastewater monitoring, correspond with shifts in audience profiles of concern over time.

## 5. Conclusions

This study examined the risk perception profiles and associated factors linked to different audience segments regarding wastewater monitoring for COVID-19. The identification of four distinct groups—the *worrisome*, the *practical*, the *community-oriented*, and the *minimally concerned*—emphasizes the need for a nuanced understanding of public attitudes toward health surveillance technologies. Covariate analysis revealed that communal coping orientation, attitudes toward monitoring, misinformation beliefs, and knowledge levels were significantly associated with profile membership, offering strategic entry points for tailored communication efforts. Additionally, demographic characteristics such as age, education, and political ideology further underscore the importance of nuanced approaches to message design. Together, these findings provide valuable insights for developing targeted public health communication strategies that enhance understanding and acceptance of health surveillance technologies, ultimately supporting efforts to promote public health.

## Figures and Tables

**Table 1:** Demographic information of the participants (*N* = 322).

	Study Participants	Colorado Residents
Variable	%	
**Sex**		
Female	50.0	49.4
Ethnicity *		
White	83.1	84.5
Black or African American	3.3	5.8
Asian	4.2	5.1
Hispanic, Latino, or Spanish origin	6.6	22.7
American Indian or Alaska native	3.3	3.5
Native Hawaiian or Pacific Islander	0.9	0.4
Some other race, ethnicity, or origin	2.1	16.7
**Education**		
Eighth grade or less	0.3	4.6
Some high school or less	1.0	5.6
Graduated high school	20.7	44.8
Associate’s degree	8.9	8.8
Bachelor’s degree	48.7	21.8
Graduate or professional degree	20.4	14.3
**Income**		
Less than $35,000	17.6	16.7
$35,000 to less than $50,000	14.7	8.5
$50,000 to less than $75,000	28.8	15.1
$75,000 to less than $100,000	17.3	13.0
Over $100,000	21.7	46.8

Note. * These values sum to more than 100% because the categories are not mutually exclusive.

**Table 2:** Risk perceptions about wastewater monitoring for COVID-19: Indicators for latent class analysis (*N* = 322).

Indicators (I Am Concerned That Wastewater Monitoring…)	Code	Label	*n*	%
1. may not provide accurate early warnings of potential COVID-19 outbreaks.	1	Not concerned	102	31.7
	2	Concerned or Unsure	220	68.3
2. may not be cost-effective	1	Not concerned	86	26.7
	2	Concerned or Unsure	236	73.3
3. may be used to support drastic measures such as shutting down businesses and schools.	1	Not concerned	90	28
	2	Concerned or Unsure	232	72
4. may be used to imply that certain individuals or communities are responsible for spreading the coronavirus.	1	Not concerned	110	34.2
	2	Concerned or Unsure	212	65.8
5. may contribute to inequitable resource allocation in coping with COVID-19.	1	Not concerned	103	32
	2	Concerned or Unsure	219	68
6. may contribute to stigmatizing certain individuals or communities.	1	Not concerned	112	34.8
	2	Concerned or Unsure	210	65.2
7. may let my health information be readily available to people/organizations unauthorized to view or work with the data	1	Not concerned	138	42.9
	2	Concerned or Unsure	184	57.1
8. may be used to trace the use of illegal materials, such as opioids and other drugs.	1	Not concerned	141	43.8
	2	Concerned or Unsure	181	56.2

**Table 3:** Model fit and diagnostic information for comparison of latent class models.

Model Fit Criteria								
Number of Classes	LL	G^2^		df	AIC	BIC		Adjusted BIC
2	−1287.35	301.74		238	335.74	399.91		345.99
3	−1256.10	239.24		229	291.24	389.38		306.91
**4**	−1239.23	**205.51**		**220**	**275.51**	**407.62**		**296.60**
5	−1229.97	187		211	275.00	441.08		301.51
6	−1221.42	172.09		202	278.09	478.14		310.03
7	−1211.07	149.20		193	273.20	507.22		310.56
**Diagnostic criteria**								
Number of classes	Smallest class count (*n*)		Smallest class size (%)		Entropy		Average latent class posterior probability	
2	134		41.56		0.87		0.96	
3	71		22.11		0.78		0.90	
**4**	**57**		**11.37**		**0.81**		**0.90**	
5	26		7.92		0.77		0.87	
6	15		4.65		0.76		0.88	
7	17		5.31		0.79		0.85	

**Table 4:** Item-response probabilities for a four-class model given latent class membership.

I Am Concerned That Wastewater Monitoring…	Minimally Concerned (22%)	Practical (19%)	Community -Oriented (11%)	Worrisome (48%)
1. may not provide accurate early warnings of potential COVID-19 outbreaks.	0.23	**0.73**	0.33	**0.96**
	(0.06)	**(0.10)**	(0.11)	**(0.02)**
2. may not be cost-effective	0.31	**0.99**	0.19	**0.96**
	(0.08)	**(0.04)**	(0.23)	**(0.02)**
3. may be used to support drastic measures such as shutting down businesses and schools.	0.31	**0.64**	**0.65**	**0.96**
	(0.06)	**(0.09)**	**(0.10)**	**(0.02)**
4. may be used to imply that certain individuals or communities are responsible for spreading the coronavirus.	0.16	0.39	**0.79**	**0.97**
	(0.06)	(0.10)	**(0.11)**	**(0.02)**
5. may contribute to inequitable resource allocation in coping with COVID-19.	0.07	**0.70**	**0.66**	**0.96**
	(0.04)	**(0.10)**	**(0.12)**	**(0.02)**
6. may contribute to stigmatizing certain individuals or communities.	0.10	0.42	**0.85**	**0.96**
	(0.05)	(0.11)	**(0.09)**	**(0.02)**
7. may let my health information be readily available to people/organizations	0.07	0.29	0.48	**0.94**
	(0.04)	(0.09)	(0.11)	**(0.02)**
8. may be used to trace the use of illegal materials, such as opioids and other drugs.	0.09	0.31	**0.58**	**0.88**
	(0.05)	(0.08)	**(0.11)**	**(0.03)**

Notes. Percentages reflect the number of participants likely to be in each profile. Cells contain the likelihood of being concerned or unsure of the given risk perception. Likelihoods greater than 50% appear in bold.

**Table 5:** Covariate analysis with the minimally concerned as the reference group.

	Practical			Community-Oriented			Worrisome		
	OR	95% CI	*p*	OR	95% CI	*p*	OR	95% CI	p
Model 1									
Communal coping	0.14	[0.03, 0.64]	0.011	0.43	[0.10, 1.79]	0.247	0.37	[0.07, 1.83]	0.221
COVID-19 misinfo	3.38	[0.74, 15.44]	0.116	7.93	[1.75, 5.96]	0.007	84.83	[4.35, 1654.3]	0.003
WW knowledge	3.37	[0.067, 2.080]	0.261	0.18	[0.04, 0.91]	0.038	0.07	[0.01, 0.53]	0.010
WW attitude	0.16	[0.041, 0.632]	0.009	0.07	[0.02, 0.30]	<0.001	0.16	[0.03, 0.97]	0.046
Model 2									
Age	0.98	[0.946, 1.022]	0.393	1.00	[0.97, 1.04]	0.802	0.96	[0.93, 0.98]	0.003
Education	0.55	[0.331, 0.929]	0.025	1.11	[0.72, 1.73]	0.633	0.98	[0.69, 1.38]	0.894
Gender	0.64	[0.195, 2.112]	0.465	1.14	[0.50, 2.61]	0.762	0.85	[0.42, 1.74]	0.665
White	4.48	[0.43, 48.47]	0.217	0.71	[0.18, 2.74]	0.616	0.53	[0.17, 1.59]	0.257
Political ideology	2.40	[1.46, 3.96]	<0.001	1.05	[0.69, 1.62]	0.812	2.89	[1.99, 4.18]	<0.001

*Note*. The table reflects the relation between each covariate and the probability of latent class membership (e.g., odds) versus membership in the minimal concerns group. OR = Odds ratio; WW = Wastewater monitoring.

## Data Availability

The data that support the findings of this study are available on request from the corresponding author.

## Supplementary Materials

The following supporting information can be downloaded at https://www.mdpi.com/article/10.3390/ijerph22121782/s1. Supplementary Material S1: Informative message about wastewater monitoring and survey items; Supplementary Material S2: Distribution of participant responses to concerns with wastewater monitoring for COVID-19.
