# Supplementary material for: Communicating Community-Based Public Health Surveillance: Lessons from Profiling Public Risk Perceptions of COVID-19 Wastewater Monitoring
Source: Int J Environ Res Public Health. 2025 Nov 25;22(12):1782. doi: 10.3390/ijerph22121782 (PMC12733286; doi:10.3390/ijerph22121782)
Supplement: Supplementary file 1 [file ijerph-22-01782-s001.zip › ijerph-3950551-supplementary.pdf]

Supplementary Material S1

Informative message about wastewater monitoring and survey items

To answer the following questions, please read the following message about the COVID-19 monitoring in wastewater carefully.

People infected with SARS-CoV-2 can shed the virus in their feces, even if they don't have symptoms. The virus can then be detected in wastewater, enabling wastewater surveillance to capture the presence of SARS-CoV-2 shed by people with and without symptoms. This allows wastewater surveillance to serve as an early warning that COVID-19 is spreading in a community. Since August 2020, the state of Colorado has been collaborating with labs and wastewater utilities statewide to test for COVID-19 virus particles in wastewater.

A graphic description of the wastewater monitoring process is as follows:

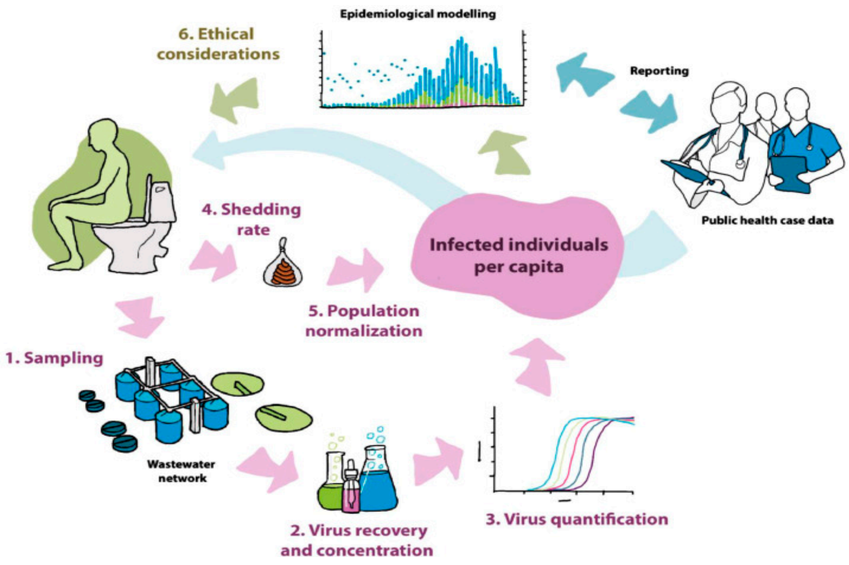

Survey Items

Communal coping orientation

Thinking about how you and your local community have been handling COVID-19, please indicate the extent to which you agree with the following statements (1 = *Strongly disagree*, 2 = *disagree*, 3 = *neither disagree nor agree*, 4 = *agree*, 5 = *strongly agree*).

- When I think about COVID-19, I mostly think about…
    - how it is my community's health issue that we face together
    - how it influences my community members' lives
    - how my community is affected by it.
- It is my community's responsibility to prevent the spread of COVID-19.
- My community members take an active role in adapting to a new normal following COVID-19.
- My community members rely on one another to cope with COVID-19.

COVID-19 misinformation

Please indicate the extent to which you agree with each statement below (1 = *Strongly disagree*, 2 = *disagree*, 3 = *neither disagree nor agree*, 4 = *agree*, 5 = *strongly agree*)

- Coronavirus is probably a hoax.
- Public health authorities are exaggerating the seriousness of coronavirus.
- Information about treatments for coronavirus is being suppressed by those who want the pandemic to continue.
- The coronavirus is not more dangerous than the seasonal flu.
- Coronavirus was created in a lab.

Knowledge about COVID-19 monitoring in wastewater
Please indicate the *true (1)* or *false* (2) to each statement.

- COVID-19 could be detected in wastewater (True).
- Monitoring wastewater could determine which person or persons in a household have COVID-19 (False).
- Measuring the level of the virus in wastewater is a faster way to detect COVID-19 in a community than testing everyone in the community (False).

Attitudes toward COVID-19 monitoring in wastewater
Please indicate the extent to which you agree with each statement below (1 = *Strongly disagree*, 2 = *disagree*, 3 = *neither disagree nor agree*, 4 = *agree*, 5 = *strongly agree*).

- I like the idea of COVID-19 monitoring in wastewater.
- I think COVID-19 monitoring in wastewater is desirable.
- I think COVID-19 monitoring in wastewater is good.
- I am positive toward the idea of COVID-19 monitoring in wastewater.

Risk beliefs
Please indicate the extent to which you agree with each statement below in thinking about COVID-19 monitoring in wastewater (1 = *Strongly disagree*, 2 = *disagree*, 3 = *neither disagree nor agree*, 4 = *agree*, 5 = *strongly agree*)

- I am concerned that COVID-19 monitoring in wastewater
  - may not provide accurate early warnings of potential COVID-19 outbreaks.
  - may not be cost-effective.
  - may be used to support drastic measures such as shutting down businesses and schools.
  - may be used to imply that certain individuals or communities are responsible for spreading the coronavirus.
  - may contribute to inequitable resource allocation in coping with COVID-19.
  - may contribute to stigmatizing certain individuals or communities.
  - may let my health information be readily available to people/organizations unauthorized to view or work with the data.
  - may be used to trace the use of illegal materials, such as opioids and other drugs.

Demographic variables

- How old are you? ______

- Which racial and ethnic categories do you identify with? Please mark all boxes that apply.
  - White

- Hispanic, Latino, or Spanish origin
- Black or African American
- Asian
- American Indian or Alaska Native
- Native Hawaiian or other Pacific Islander
- Some other race, ethnicity, or origin

- What is your sex?
    - Female
    - Male
    - Transgender
    - Prefer not to answer

- What is the highest degree of level of school that you have completed?
    - Eighth grade or less
    - Attended high school
    - Graduated from high school
    - Graduated from college with an associate's degree (AA, AS, etc.)
    - Graduated from college with a bachelor's degree (BA, BS, etc.)
    - Graduated with a graduate degree (MA, JD, MD, PhD, etc.)

- Please indicate your political ideology (1= *extremely liberal*, 2 = *slightly liberal*, 3 = *moderate or middle of the road*, 4 = *slightly conservative*, 5 = *extremely conservative*)

- Are you currently…?
    - Employed by a company that you do not own
    - Self-employed
    - Full-time student
    - Unemployed
    - Retired

- Approximately, what is your annual household income?
    - Less than $20,000
    - $20,000 to less than $35,000
    - $35,000 to less than $50,000
    - $50,000 to less than $75,000
    - $75,000 to less than $100,000
    - Over $100,000

Supplementary Material S2

Supplementary Table S1. Distribution of participant responses to concerns with wastewater monitoring for COVID-19

Indicators (I am concerned that wastewater monitoring…)	1	2	3	4	5	*M*	*SD*
1. may not provide accurate early warnings of potential COVID-19 outbreaks.	41	61	81	98	41	3.11	1.23
2. may not be cost-effective	38	48	72	103	61	3.31	1.27
3. may be used to support drastic measures such as shutting down businesses and schools.	40	50	76	100	56	3.25	1.26
4. may be used to imply that certain individuals or communities are responsible for spreading the coronavirus.	47	63	62	108	42	3.11	1.28
5. may contribute to inequitable resource allocation in coping with COVID-19.	47	56	79	97	43	3.10	1.26
6. may contribute to stigmatizing certain individuals or communities.	47	65	47	109	54	3.18	1.33
7. may let my health information be readily available to people/organizations unauthorized to view or work with the data	80	58	63	84	37	2.81	1.37
8. may be used to trace the use of illegal materials, such as opioids and other drugs.	78	63	62	86	33	2.79	1.34

Note. This table presents the full distribution of participant responses to survey items assessing concerns about wastewater monitoring for COVID-19. Each number under columns 1–5 indicates the number of participant responses. Response options were measured on a five-point scale: 1 = *Strongly Disagree*, 2 = *Somewhat Disagree*, 3 = *Neither Disagree nor Agree*, 4 = *Somewhat Agree*, and 5 = *Strongly Agree*. *M* = *Mean*; *SD* = *Standard Deviation*.
